# The “Futile Labour” Paradox: occupational physical activity fails to offset diabetes burden and is associated with microvascular stress signals in rural older adults

**DOI:** 10.3389/fpubh.2026.1790766

**Published:** 2026-03-16

**Authors:** Kefeng Zhang, Xiaohui Liu, Chi Ma, Yongheng Zhao, Xuefeng Xi, Gaixia Hou, Limeng Liu, Yan Gao, Dehui Zhang

**Affiliations:** 1 Department of Physical Education and Military Training, East University of Heilongjiang, Harbin, China; 2 School of Physical Education, Suihua University, Suihua, China; 3 College of Physical Education and Health, Mudanjiang Normal University, Mudanjiang, China; 4 School of Wushu, Henan University, Kaifeng, China; 5 Institute of Aging Studies, Henan University, Kaifeng, China; 6 School of Physical Education, Jiamusi University, Jiamusi, China; 7 Wangkui County East City Community Health Service Center, Suihua, China

**Keywords:** occupational physical activity, renal microvascular stress, rural ageing, total diabetes burden, triglyceride–glucose (TyG) index, undiagnosed hyperglycemia

## Abstract

**Background:**

Physical activity is widely promoted as a cornerstone of healthy ageing; however, this assumption largely derives from leisure-time exercise and may not apply to labour-dependent older populations. In rural settings, occupational physical activity (OPA) is often necessity-driven, prolonged, and embedded within structural constraints, yet its associations with metabolic and vascular outcomes in later life remain poorly understood.

**Methods:**

We conducted a community-based cross-sectional study of 2,258 adults aged ≥65 years from a representative agricultural region in Northeast China. Labour-type physical activity was classified as inactive, moderate, or high intensity. To mitigate underdiagnosis, we defined total diabetes burden as either diagnosed diabetes or undiagnosed hyperglycemia. Systemic insulin resistance was assessed using the triglyceride–glucose (TyG) index. Urine occult blood (UOB) positivity was analyzed as an exploratory marker compatible with physiological stress. Associations were examined using modified Poisson regression with robust variance, robust linear regression, and prespecified sensitivity analyses.

**Results:**

High-intensity labour was associated with a modest reduction in insulin resistance among individuals without diagnosed diabetes (*β* = −0.079, *p* = 0.005) and a lower prevalence of undiagnosed hyperglycemia (PR 0.69, 95% CI 0.53–0.88). In contrast, high-intensity labour was more common among individuals with diagnosed diabetes (PR 1.43, 95% CI 1.11–1.85). These opposing associations did not show a significant net association with total diabetes burden (PR 0.99, *p* = 0.912). Independently of metabolic status, high-intensity labour was associated with a higher prevalence of UOB positivity (PR 1.25, 95% CI 1.09–1.44).

**Conclusion:**

In labour-dependent rural older adults, heavy occupational labour may not confer metabolic benefit at the population level and is independently associated with a microvascular stress–related signal. This “Futile Labour Paradox” challenges the assumption that physical activity is uniformly beneficial in later life and underscores the need to distinguish occupational labour from discretionary exercise.

## 1 Introduction

Population ageing poses a growing global public health challenge, with chronic metabolic diseases representing a major threat to functional independence, quality of life, and healthy longevity in later adulthood. Regular physical activity is widely promoted as a cornerstone of healthy ageing and is consistently associated with improved metabolic control, cardiovascular health, and reduced mortality risk (1, 2). Accordingly, public health guidelines frequently advocate increased physical activity as a near-universal preventive strategy for older adults.

However, accumulating evidence indicates that not all forms of physical activity confer equivalent health benefits. While leisure-time physical activity (LTPA) is robustly associated with favorable metabolic and cardiovascular outcomes, occupational physical activity (OPA) has demonstrated heterogeneous—and in some cases adverse—health associations, a phenomenon often described as the “physical activity paradox” (3–5). Importantly, much of the existing literature is derived from working-age, urban, or industrialized populations, leaving labour-dependent rural older adults—whose physical exertion is frequently necessity-driven rather than discretionary—substantially underrepresented in current evidence frameworks.

Interpretation of labour-related metabolic risk is further complicated by the pervasive underdiagnosis of diabetes in rural and resource-limited settings. Undiagnosed hyperglycemia remains common among older adults with restricted access to routine healthcare, raising concern that studies relying solely on physician-diagnosed diabetes may underestimate total metabolic burden and distort exposure–outcome relationships (6–8). In labour-intensive agricultural contexts, continued physical work may both mask early symptoms and transiently influence glucose dynamics, making it methodologically essential to distinguish between diagnosed and undiagnosed disease states when evaluating the health implications of occupational labour.

Beyond traditional metabolic outcomes, the broader biological cost of sustained occupational labour in later life remains insufficiently examined. Unlike structured exercise, high-intensity agricultural labour is typically characterized by prolonged duration, repetitive physical strain, limited recovery opportunity, and inadequate nutritional compensation. These features suggest that, particularly among older adults, heavy labour may function not merely as a metabolic stimulus but as a chronic physiological stressor, with potential consequences extending to microvascular integrity—an underexplored dimension of labour-related health risk (9–12). Importantly, in cross-sectional population datasets, urinary dipstick abnormalities should be interpreted as non-specific signals compatible with physiological or urogenital stress rather than as definitive evidence of clinical microvascular injury (13, 14).

To frame these observations within established epidemiological theory while remaining explicit about the limits of causal inference, we introduce three closely related, hypothesis-generating constructs. First, we use “Futile Labour Paradox” to describe a pattern in which higher labour-related physical activity shows a non-protective net association with metabolic burden at the population level, consistent with the physical activity paradox framework (3–5). Second, we define the “Sick Labourer Phenotype” as the possibility that individuals with poorer underlying health may remain in high-labour exposure due to economic or role constraints, conceptually aligned with healthy/ill-worker selection processes (15, 16). Third, we use “Medical Offloading” to refer to public-health strategies that reduce non-discretionary physical burden (e.g., heavy manual labour) as a complement to conventional activity-promotion approaches in labour-dependent older adults, consistent with life-course and structural-health perspectives (17, 18).

To address these gaps, we utilised a large, community-based cohort from a representative agricultural region of Northeast China to examine cross-sectional associations between labour intensity and complementary metabolic and stress-related endpoints. Using a standardised single-item frequency-based labour measure as a pragmatic proxy for occupational activity in routine public-health data, we evaluated labour intensity in relation to (1) total diabetes burden (capturing both diagnosed and undiagnosed disease), (2) the triglyceride–glucose (TyG) index as an indicator of insulin resistance, and (3) urine occult blood positivity as an exploratory, non-specific urinary abnormality marker. We emphasise that this study is designed to characterise associations and generate context-specific hypotheses rather than to infer temporality or causality. By integrating metabolic, mechanistic, and urinary-abnormality dimensions within a labour-dependent ageing population, this study aims to provide a more comprehensive and context-sensitive assessment of occupational physical activity as a health exposure in later life.

## 2 Methods

### 2.1 Study design and participants

This community-based, cross-sectional study utilized real-world data from the 2025 National Basic Public Health Service Program (annual community health examination cycle) in Wangkui County, Heilongjiang Province, a representative cold-climate agricultural region in Northeast China. The study design, data collection, and reporting strictly adhered to the STROBE (Strengthening the Reporting of Observational Studies in Epidemiology) guidelines (19).

Ethical approval was granted by the Ethics Committee of the School of Medicine, Henan University (Approval No. HUSOM2025-929), in accordance with the Declaration of Helsinki. Written informed consent was obtained from all participants prior to data collection.

The study population comprised permanent community-dwelling residents aged 65 years or older. Inclusion criteria were: (i) age ≥65 years; (ii) completion of the standardized assessment protocol, including questionnaire, physical examination, and fasting biochemical tests; and (iii) provision of informed consent. Exclusion criteria were applied to ensure data integrity and analytic validity, including: (i) missing key metabolic measurements (e.g., fasting plasma glucose or lipid profiles); (ii) biologically implausible anthropometric values suggestive of recording error; and (iii) indeterminate diabetes status (i.e., absence of self-reported diabetes history combined with missing glycemic data).

Specifically, biologically implausible anthropometric values were screened using pre-specified plausibility ranges (adult/older-adult field QC rules): height <120 cm or >200 cm, weight <30 kg or >150 kg, or BMI < 10 or >60 kg/m^2^; any records outside these ranges were excluded as likely recording errors.

After pre-specified data cleaning procedures and quality control of the triglyceride–glucose (TyG) index, the final analytic sample consisted of 2,258 older adults. TyG quality control removed non-finite values and extreme outliers outside a physiologically plausible range (TyG < 4 or >13), consistent with prior epidemiological practice for preventing leverage by data-entry errors or assay anomalies.

**Figure 1:**
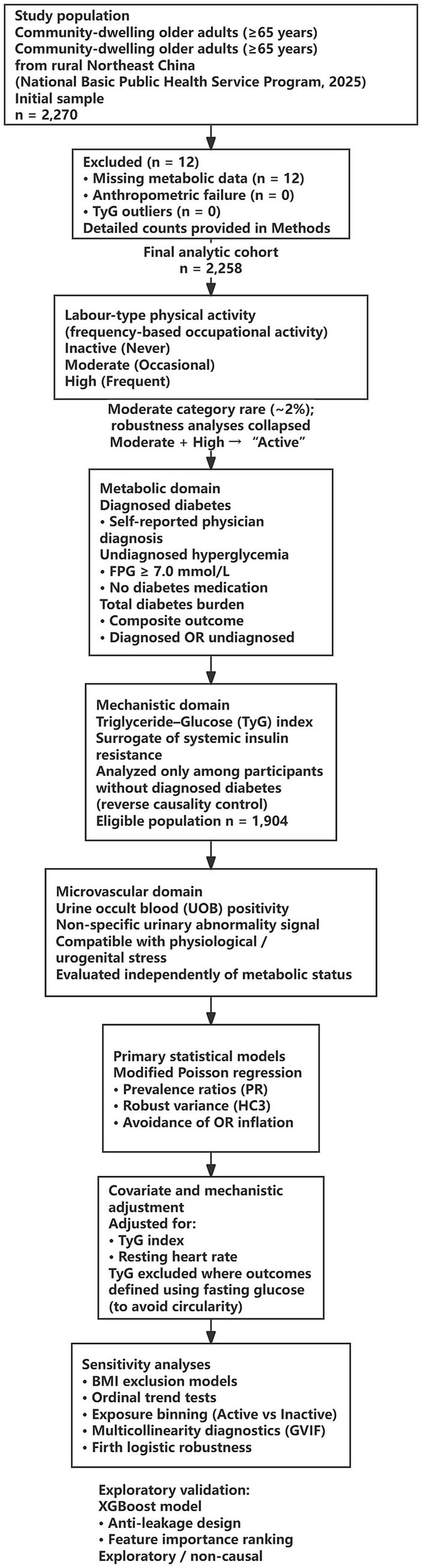
Study design and analytical framework. Flowchart of participant selection, exposure classification, outcomes, and analyses.

Figure 1 provides an overview of participant selection, exposure classification, outcome domains, and the overall analytical framework of the study.

### 2.2 Data collection and measurements

*Anthropometry and vital signs*: Height and weight were measured using calibrated instruments. Body mass index (BMI) was calculated as weight (kg) divided by height squared (m^2^). Resting blood pressure was measured using an automated sphygmomanometer (Omron HBP-9020) after at least 5 min of seated rest; the mean of two readings was recorded. If the two readings differed materially, a repeat measurement was performed per routine examination workflow and the mean of the two closest readings was recorded (program QC practice). Resting heart rate, derived from a standard 12-lead electrocardiogram (ECG), was utilized as a physiological proxy for sympathetic autonomic tone (20).

*Biochemical assays*: Venous blood samples were collected after an overnight fast of >8 h. Serum concentrations of fasting plasma glucose (FPG), triglycerides (TG), total cholesterol (TC), and lipid fractions (LDL-C, HDL-C) were analyzed using an automated biochemistry analyzer (Beckman AU5821).

*Urinalysis*: Urine occult blood was assessed using standardized dipstick urinalysis. Urine occult blood positivity was defined as any dipstick-detected blood (trace or higher), and was analyzed as a binary outcome (positive vs. negative).

For analytic robustness, dipstick outputs recorded as symbols/strings (e.g., “trace,” “+,” “++,” “positive”) were harmonized into a strict binary coding (0/1) prior to modeling; records with unresolvable/ambiguous coding were treated as missing for this endpoint.

### 2.3 Operational definitions of variables

#### 2.3.1 Exposure: labour-type physical activity

Physical activity was assessed using a standardized frequency-based item. Given the specific rural agricultural context, the frequency-based item is widely interpreted as reflecting labour-intensive agricultural/occupational workloads rather than structured leisure-time exercise. Consequently, participants were categorized into three intensity groups: Inactive (Never), Moderate (Occasional), and High (Frequent).

Because the moderate category was rare (~2%), we pre-specified an additional robustness analysis that collapsed Moderate and High into a binary “Active” category (Ex_Bin: Active vs. Inactive) to evaluate the stability of main conclusions under small-stratum conditions.

We explicitly acknowledge that this single-item frequency proxy does not provide metabolic equivalent task (MET) values and may introduce non-differential misclassification; therefore, effect estimates should be interpreted as associations with “labour-frequency exposure” rather than calibrated energy expenditure.

#### 2.3.2 Primary outcome: total diabetes burden

To mitigate the “iceberg bias” of undiagnosed cases prevalent in rural areas, Total Diabetes was defined as a composite outcome meeting either of the following criteria: (1) a self-reported history of physician-diagnosed diabetes; or (2) a fasting plasma glucose (FPG) level ≥7.0 mmol/L at the time of examination. This composite definition was used to capture the total diabetes burden and reduce misclassification due to underdiagnosis in rural settings.

Diagnosed diabetes was additionally cross-referenced against hypoglycemic medication indicators when available, and undiagnosed hyperglycemia was defined only among participants without diagnosed diabetes and without hypoglycemic medication use.

#### 2.3.3 Mechanistic indicator: TyG index

The Triglyceride–Glucose (TyG) index, a validated surrogate marker for systemic insulin resistance, was calculated as (21):

TyGIndex=ln[(Triglycerides(mg/dL)×FPG(mg/dL))/2]

Triglycerides and fasting plasma glucose (FPG) were converted from mmol/L to mg/dL prior to calculation (triglycerides × 88.57; FPG × 18).

To prevent definitional overlap (“circularity”), TyG was never included as a covariate in models whose outcomes were defined using contemporaneous FPG thresholds (i.e., undiagnosed hyperglycemia and total diabetes).

### 2.4 Statistical analysis

All statistical analyses were conducted using R (version 4.5.0; R Foundation for Statistical Computing). Continuous variables were compared using Student’s *t*-tests or ANOVA as appropriate, and categorical variables were compared using Chi-square tests.

*Effect measure and primary models (prevalence ratios)*: Given the high prevalence of diabetes in this older cohort (>10%), odds ratios from logistic regression may overestimate the relative risk. Therefore, we used modified Poisson regression with robust (HC3) standard errors to estimate prevalence ratios (PRs) and 95% confidence intervals (CIs) for all primary cross-sectional associations.

*Covariate adjustment*: Model 1 (Base Model) adjusted for age, sex, body mass index (BMI), systolic blood pressure (SBP), and health-related behaviors (smoking status, drinking frequency, and diet type, where available). Model 2 (Mechanistic Model) additionally adjusted for the triglyceride–glucose (TyG) index and resting heart rate, to evaluate whether persistent insulin resistance and physiological stress could account for the observed non-protective metabolic pattern of labour exposure.

*Avoiding definitional overlap*: To avoid mathematical circularity, TyG was included only in models whose outcomes were not defined using contemporaneous fasting plasma glucose values (i.e., diagnosed diabetes).

*Missing data and prespecified robustness analyses*: Analyses were performed using complete-case data; therefore, sample sizes could vary by model due to missing covariates (primarily lifestyle variables and urine dipstick markers). To address reviewer concerns regarding (i) the very small moderate-intensity stratum (~2%), (ii) potential multicollinearity involving TyG-related constructs, and (iii) effect-measure consistency for common outcomes, we conducted the following prespecified supplementary analyses:

1. We re-estimated all primary diabetes models after collapsing Moderate and High into a single “Active” category (Ex_Bin: Active vs. Inactive).
2. We evaluated multicollinearity using variance inflation factors (VIF/GVIF) in a linear model with TyG as the dependent variable and the mechanistic-model covariate set as predictors, after excluding any zero-variance covariates in the complete-case subset.
3. For urine occult blood (UOB) positivity, we estimated PRs using modified Poisson regression with robust (HC3) standard errors as the primary analysis, and reported logistic regression odds ratios (ORs) as sensitivity analyses for comparability.

## 3 Results

### 3.1 Study population and diabetes burden

After strict data cleaning and TyG quality control, the final analytic sample comprised 2,258 older adults aged ≥65 years. The overall prevalence of total diabetes burden (composite outcome) was 22.98%. When decomposed, diagnosed diabetes accounted for 12.44%, while undiagnosed hyperglycemia among medication-free participants accounted for 12.04% (Supplementary Table S1), underscoring a substantial burden of hidden disease in this rural older adult population. Regression analyses were performed using complete-case data; thus, the effective sample size varied slightly across models depending on covariate completeness. To address reviewer concerns regarding small-stratum instability, we additionally conducted exposure binning (Ex_Bin: Active vs. Inactive) by merging the Moderate and High categories; correct reclassification was verified using an Ex_Cat × Ex_Bin cross-tabulation (Supplementary Table S6).

### 3.2 Baseline characteristics by total diabetes status

As shown in Table 1, participants with total diabetes (*n* = 519) exhibited a markedly different metabolic phenotype compared with those without diabetes (*n* = 1,739). The diabetes group had a significantly higher body mass index (25.27 vs. 24.28 kg/m^2^; *p* < 0.001) and a substantially elevated TyG index (9.38 vs. 8.83; *p* < 0.001), with a large standardized mean difference (SMD = 0.951), indicating profound systemic insulin resistance.

**Table 1:** Baseline characteristics of the study population by total diabetes status.

Variable	Total diabetes (*n* = 519)	No diabetes (*n* = 1,739)	*p*-value	SMD
Age, years	71.86 ± 5.23	72.96 ± 6.18	<0.001	0.191
Men, *n* (%)	258 (49.7)	844 (48.5)	0.674	0.024
BMI, kg/m^2^	25.27 ± 3.31	24.28 ± 3.31	<0.001	0.300
TyG index	9.38 ± 0.62	8.83 ± 0.53	<0.001	0.951
Resting heart rate, bpm	74.77 ± 11.35	73.44 ± 33.28	0.373	0.053
Systolic BP, mmHg	146.49 ± 21.47	145.27 ± 20.44	0.239	0.058
Labour intensity, *n* (%)			0.054	—
Inactive	171 (33.9)	674 (39.7)		
Moderate	10 (2.0)	36 (2.1)		
High	324 (64.2)	988 (58.2)		
Urine occult blood positive, *n* (%)	148 (28.5)	560 (32.2)	0.125	0.080

Values are presented as mean ± standard deviation or *n* (%). *p*-values were derived from Student’s *t*-test for continuous variables and Chi-square tests for categorical variables. SMD, standardized mean difference; BMI, body mass index; TyG, triglyceride–glucose index; BP, blood pressure. The moderate-intensity labour category was rare (~2%, *n* = 46), reflecting the polarized distribution of rural agricultural workloads. Accordingly, moderate-versus-inactive contrasts in regression analyses are statistically imprecise and should be interpreted with caution. To address small-stratum instability, prespecified robustness analyses were conducted by collapsing moderate and High into a binary “Active” group (Ex_Bin). These analyses yielded directionally consistent results across all primary outcomes (Supplementary Tables S7–S10), indicating that the main conclusions are not driven by sparse data in the moderate-intensity stratum. Resting heart rate exhibited substantial variability, likely reflecting arrhythmia prevalence and ECG-derived measurement heterogeneity in this older adult cohort.

Participants with diabetes were slightly younger (71.9 vs. 73.0 years; *p* < 0.001), while sex distribution was comparable between groups (*p* = 0.674). Notably, the distribution of labour intensity differed marginally (*p* = 0.054), with a higher proportion of high-intensity labour observed in the diabetes group (64.2%) compared with the non-diabetes group (58.2%), challenging the conventional “healthy worker” assumption. Urine occult blood positivity did not differ significantly in unadjusted comparisons (*p* = 0.125). Given the hypothesis of labour-related microvascular strain, this marker was further evaluated in multivariable models (Section 3.5). As prespecified, the moderate-intensity labour category was rare (~2%); therefore, Moderate-versus-Inactive estimates are interpreted as imprecise, and stable inference for the overall exposure contrast is additionally supported by Ex_Bin robustness models (Supplementary Tables S7–S10).

### 3.3 Labour intensity and insulin resistance (TyG) in the non-diagnosed population

To minimize reverse causality driven by post-diagnosis lifestyle modification, we evaluated the association between labour intensity and the TyG index exclusively among participants without diagnosed diabetes or hypoglycemic medication (n = 1,904).

**Figure 2:**
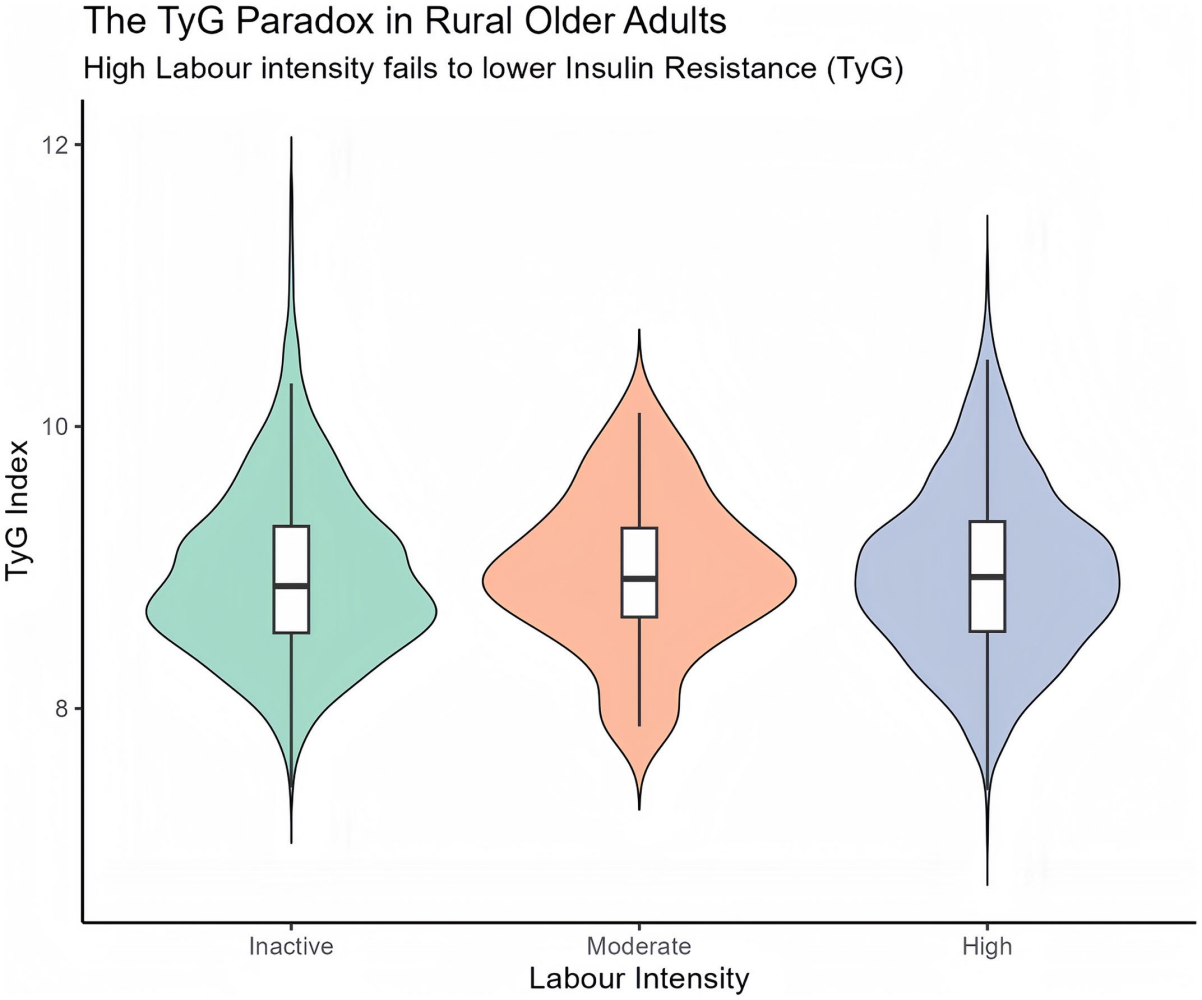
Distribution of the triglyceride–glucose (TyG) index across categories of labor intensity among older adults without diagnosed diabetes. Violin plots depict the kernel density distribution of TyG values, with embedded boxplots indicating the median and interquartile range. In robust linear regression adjusted for demographic and cardiometabolic covariates (Table 2), high-intensity labor was associated with a modest reduction in TyG index compared with inactivity. However, absolute TyG levels remained elevated across all labor categories, indicating that although heavy labor confers limited physiological improvement in insulin resistance, the magnitude of this reduction is insufficient to substantially alleviate the overall metabolic burden.

In robust linear regression, high-intensity labour was associated with a modest reduction in TyG index compared with inactivity (*β* = −0.079; 95% CI − 0.134 to −0.024; *p* = 0.005), whereas moderate labour intensity showed no significant association (*p* = 0.550) (Table 2). As illustrated in Figure 2, although heavy labour confers a statistically detectable metabolic benefit relative to inactivity, absolute TyG levels remained elevated across all labour categories, suggesting that this modest improvement may be insufficient to meaningfully offset the broader diabetes burden. Consistent with reviewer guidance, Figure 2 is interpreted in terms of both statistical and practical magnitude: the between-group shift is modest relative to the overall distributional overlap, supporting a limited physiological improvement interpretation rather than a clinically transformative effect.

**Table 2:** Association between labour intensity and TyG index among participants without diagnosed diabetes.

Variable	*β* coefficient	95% CI	*p*-value	*n*
Moderate vs. inactive	−0.050	−0.215 to 0.115	0.550	1,904
High vs. inactive	−0.079	−0.134 to −0.024	0.005	1,904

*β* coefficients were estimated using robust linear regression. Models were adjusted for age, sex, body mass index, systolic blood pressure, smoking status, drinking frequency, and diet type. Analyses were restricted to participants without self-reported diabetes or hypoglycemic medication use to minimize reverse causality arising from post-diagnosis behavioral modification. TyG, triglyceride–glucose index.

*Restriction and rationale*: Analyses were restricted to participants without self-reported diabetes or hypoglycemic medication use to minimize reverse causality arising from post-diagnosis behavioral modification.

### 3.4 Labour intensity and diabetes outcomes (modified Poisson regression)

#### 3.4.1 Diagnosed diabetes: the “sick labourer” effect

In the base model (n = 2,181), high-intensity labour was associated with a higher prevalence of diagnosed diabetes (PR 1.43; 95% CI 1.10–1.85; *p* = 0.007), whereas moderate labour intensity was not significantly associated (PR 0.33; *p* = 0.115) (Table 3, Model 1).

**Table 3:** Labour intensity and diagnosed diabetes: modified Poisson regression.

Variable	PR	95% CI	*p*-value	*n*
Model 1				
Moderate vs. inactive	0.33	0.08–1.31	0.115	2,181
High vs. inactive	1.43	1.10–1.85	0.007	2,181
Model 2				
Moderate vs. inactive	0.35	0.09–1.37	0.130	2,181
High vs. inactive	1.43	1.11–1.85	0.006	2,181
TyG index	1.96	1.68–2.29	<0.001	—
Resting heart rate	1.00	0.99–1.01	0.997	—

Model 1 (*n* = 2,181): base model.

Model 2 (*n* = 2,181): mechanistic model (TyG and heart rate included).

PR, prevalence ratio. Estimates were obtained using modified Poisson regression with robust variance (HC3). Model 1 adjusted for age, sex, BMI, SBP, smoking, drinking, and diet. Model 2 additionally adjusted for TyG index and resting heart rate. TyG was included only in models whose outcomes were not defined using contemporaneous fasting plasma glucose. The near-identical PR for the High vs Inactive contrast across Model 1–2 indicates minimal confounding by TyG and resting heart rate in this dataset; small differences may be obscured by rounding to two decimals.

After further adjustment for mechanistic indicators (TyG index and resting heart rate), this positive association persisted (PR 1.43; 95% CI 1.11–1.85; *p* = 0.006) (Table 3, Model 2). In this model, the TyG index emerged as a dominant risk factor for diagnosed diabetes (PR 1.96; 95% CI 1.68–2.29; *p* < 0.001), while resting heart rate showed no association. Estimates for moderate labour intensity were imprecise with wide confidence intervals and were not statistically significant. To address collinearity concerns, VIF/GVIF diagnostics indicated no problematic multicollinearity among included predictors within the complete-case subset (Supplementary Table S11). Robustness to rare-stratum instability: when Moderate and High categories were merged into a single “Active” group (Ex_Bin), the positive association with diagnosed diabetes remained (Base model PR 1.38; 95% CI 1.07–1.79; *p* = 0.014; Supplementary Table S7) and persisted in the mechanistic specification (PR 1.39; 95% CI 1.08–1.80; *p* = 0.012; Supplementary Table S8).

#### 3.4.2 Undiagnosed hyperglycemia: inverse association with high-intensity labour

Among participants free of diagnosed diabetes (n = 1,904), high-intensity labour was associated with a lower prevalence of undiagnosed hyperglycemia (PR 0.69; 95% CI 0.53–0.88; *p* = 0.004), whereas moderate labour intensity was not associated (PR 1.25; *p* = 0.504) (Table 4).

**Table 4:** Labour intensity and undiagnosed hyperglycemia among non-diagnosed participants.

Variable	PR	95% CI	*p*-value	*n*
Moderate vs. inactive	1.25	0.65–2.41	0.504	1,904
High vs. inactive	0.69	0.53–0.88	0.004	1,904

Modified Poisson regression adjusted for age, sex, BMI, hypertension, systolic blood pressure, smoking, drinking, and diet. TyG index was excluded to avoid mathematical circularity, as undiagnosed hyperglycemia is defined by fasting plasma glucose.

*Note:* The TyG index was deliberately excluded from this model to avoid mathematical circularity, as the outcome definition relies on fasting plasma glucose. This association may reflect both physiological effects and differential disease detection in labour-dependent settings.

Robustness to rare-stratum instability: in Ex_Bin models, the “Active” group similarly showed an inverse association with undiagnosed hyperglycemia (PR 0.71; 95% CI 0.55–0.91; *p* = 0.007; Supplementary Table S9).

#### 3.4.3 Total diabetes burden: the offsetting (cancellation) effect

For the composite outcome of total diabetes (n = 2,181), labour intensity showed no significant association (high vs. inactive: PR 0.99; 95% CI 0.84–1.17; *p* = 0.912) (Table 5).

**Table 5:** Labour intensity and total diabetes burden (corrected final version).

Variable	PR	95% CI	*p*-value	*n*
Moderate vs. inactive	0.80	0.46–1.40	0.441	2,181
High vs. inactive	0.99	0.84–1.17	0.912	2,181

Total diabetes was defined as diagnosed diabetes or undiagnosed hyperglycemia.

Modified Poisson regression with robust (HC3) standard errors, adjusted for age, sex, BMI, hypertension, systolic blood pressure, smoking, drinking, and diet. The TyG index was excluded to avoid mathematical circularity, as the outcome definition relies on fasting plasma glucose.

This null association reflects an offsetting effect: the inverse association between high-intensity labour and undiagnosed hyperglycemia was counterbalanced by the positive association between high-intensity labour and diagnosed diabetes (the “sick labourer” phenomenon), resulting in an overall cancellation at the population level. As a robustness check for the rare moderate stratum, Ex_Bin (Active vs. Inactive) models reproduced the same qualitative conclusion for total diabetes (PR 0.98; 95% CI 0.83–1.16; *p* = 0.843; Supplementary Table S10), supporting the stability of the “offsetting” interpretation under small-stratum conditions.

#### 3.4.4 Labour-associated urine occult blood positivity as an exploratory stress signal

In contrast to the mixed metabolic associations, labour intensity showed a consistent association with urine occult blood (UOB) positivity. This endpoint is interpreted conservatively as a **non-specific urinary abnormality compatible with urogenital/vascular stress at the population level**, rather than as definitive evidence of renal microvascular injury.

To maintain effect-measure consistency with the metabolic analyses, we estimated **prevalence ratios (PRs)** for UOB positivity using modified Poisson regression with HC3 robust standard errors based on strictly cleaned binary (0/1) UOB coding (Supplementary Table S12). High-intensity labour was associated with a higher prevalence of UOB positivity (PR 1.25; 95% CI 1.09–1.44; *p* = 0.002), whereas moderate labour intensity showed no association (PR 1.00; 95% CI 0.59–1.68; *p* = 0.999) (Supplementary Table S12).

In sex-stratified analyses, estimates were directionally consistent. For transparency, we additionally provide sex-stratified logistic-regression ORs as sensitivity analyses (Supplementary Table S2), and a direct PR-versus-OR comparison for the High vs. Inactive contrast (Supplementary Table S13). As an additional small-cell robustness check, Firth penalized logistic regression yielded a consistent estimate (OR 1.38; 95% CI 1.13–1.70; *p* = 0.002; Supplementary Table S14). Accordingly, we avoid causal language and treat this endpoint as hypothesis-generating, warranting validation using more specific renal markers (e.g., albuminuria/proteinuria and eGFR) in future studies (see Table 6).

**Table 6:** Association between labour intensity and urine occult blood positivity (exploratory stress signal).

Variable	PR	95% CI	*p*-value	*n*
Moderate vs. inactive	1.00	0.49–2.06	0.999	2,169
High vs. inactive	1.25	1.09–1.44	0.002	2,169

PRs were estimated using modified Poisson regression with robust variance (HC3). Models were adjusted for age, sex, BMI, SBP, diabetes status, TyG index (when applicable), smoking, drinking, diet type (where available), and urine dipstick protein (where available). UOB positivity was analyzed as a binary outcome and interpreted as a population-level, non-specific urinary abnormality compatible with urogenital/vascular stress rather than a disease-specific diagnosis.

### 3.5 Sensitivity analyses

*Exclusion of BMI*: To assess whether BMI acted as a mediator, models were repeated without BMI adjustment. The results were materially unchanged: high-intensity labour remained positively associated with diagnosed diabetes (PR 1.44; *p* = 0.006) and inversely associated with undiagnosed hyperglycemia (PR 0.75; *p* = 0.030) (Supplementary Tables S3–S4).

*Trend test*: When labour intensity was modeled as an ordinal variable, there was no evidence of a linear trend with total diabetes (PR per category increase 1.00; *p* = 0.946) (Supplementary Table S5), confirming a non-linear and heterogeneous association pattern.

*Summary of findings*: Collectively, these findings reveal a “Futile Labour Paradox”: while high-intensity labour confers a modest physiological protection against undiagnosed hyperglycemia, it clusters disproportionately among individuals with diagnosed diabetes, resulting in an overall null association with total diabetes burden. In addition, high-intensity labour was independently associated with higher odds of UOB positivity, a marker compatible with microvascular stress, after adjustment for metabolic status and related covariates.

**Figure 3:**
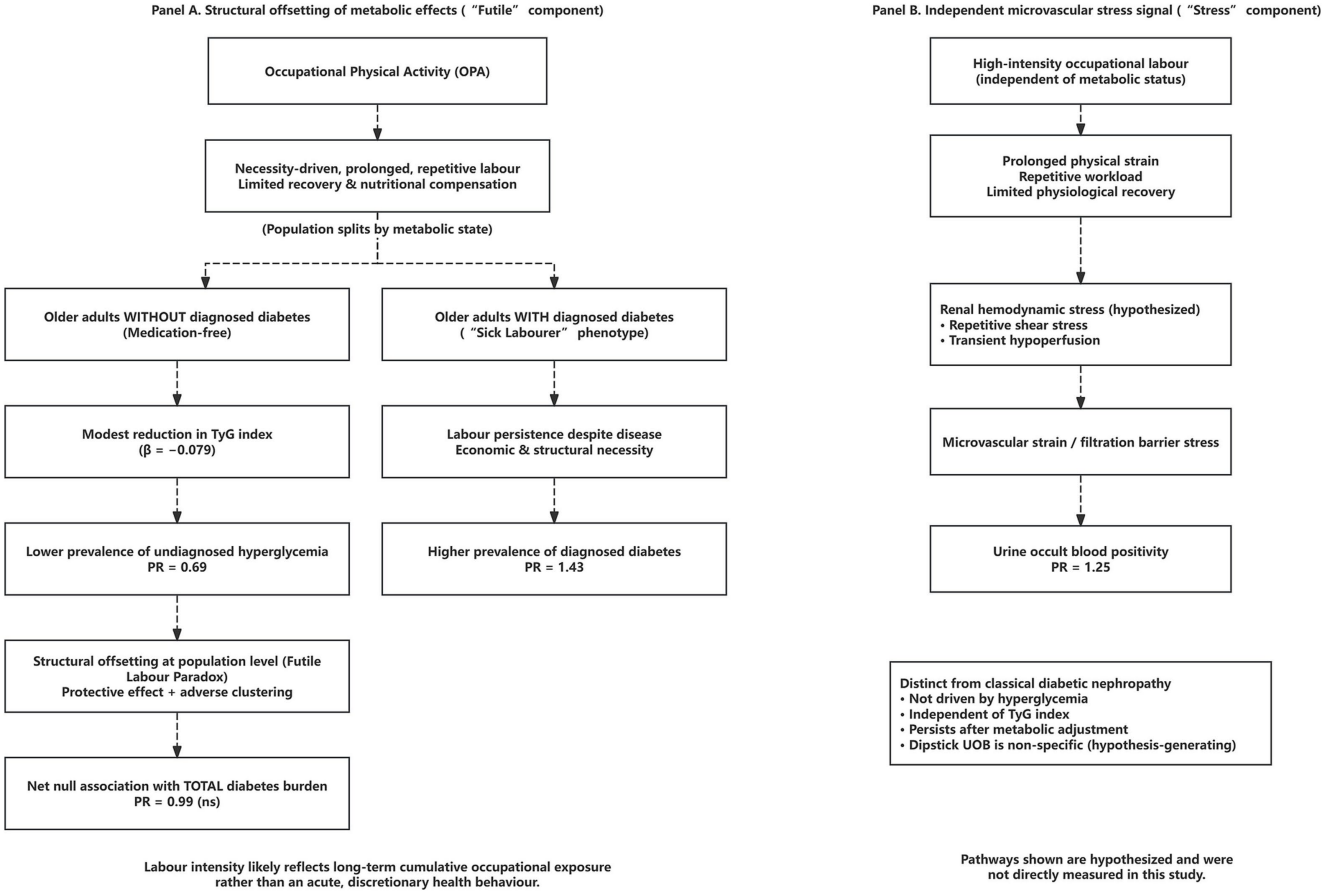
Conceptual framework of the “Futile Labour Paradox” in labour-dependent rural older adults. **(A)** Illustrates the structural offsetting of metabolic associations related to occupational physical activity (OPA). In labour-dependent rural settings, high-intensity OPA is typically necessity-driven and may involve prolonged exertion with limited recovery and nutritional compensation. Among older adults without diagnosed diabetes, high labour intensity is associated with a modest reduction in systemic insulin resistance (as indicated by the TyG index), corresponding to a lower prevalence of undiagnosed hyperglycemia. In contrast, among older adults with established diabetes (the “sick labourer” phenotype), high labour intensity clusters disproportionately due to economic and structural constraints, resulting in a higher prevalence of diagnosed diabetes. These opposing associations offset each other at the population level, yielding a net null association with total diabetes burden. **(B)** Depicts an independent association linking high-intensity occupational labour to urinary microvascular/urogenital stress signals (UOB positivity). This panel should be interpreted as a hypothesized explanatory schematic compatible with the observed labour–UOB association, rather than definitive evidence of renal microvascular injury. Dipstick occult blood positivity is non-specific and can reflect multiple potential etiologies (e.g., urogenital irritation, stones, inflammation, or transient exertional hematuria). This framework summarizes the associations observed in this study; the pathways shown are hypothesized and were not directly measured.

Taken together, these analytically independent results delineate a coherent structural pattern linking occupational labour intensity, metabolic state, and microvascular injury, which is summarized schematically in Figure 3.

## 4 Discussion

### 4.1 Reframing physical activity in later life: a stable population-level configuration under structural constraint

In this community-based cohort of older adults living in a labour-dependent rural region of Northeast China, we observed a stable population-level configuration linking occupational labour intensity, metabolic status, and a non-specific urinary abnormality marker compatible with physiological/urogenital stress. Contrary to the prevailing public health axiom that “all movement is medicine,” high-intensity occupational physical activity (OPA) was not associated with a lower overall diabetes burden (9). Instead, labour intensity displayed directionally opposing associations across metabolic states—an inverse association with undiagnosed hyperglycemia but a positive association with diagnosed diabetes—resulting in a net null association at the population level.

This null association should not be interpreted as physiological irrelevance. Rather, it reflects a structural offsetting phenomenon in which protective and adverse associations coexist and cancel within the same population. Such configurations are especially likely to emerge in ageing rural societies where labour intensity reflects constrained choice rather than discretionary health behaviour (22). Importantly, labour intensity in this setting likely serves as a proxy for long-term cumulative occupational exposure across the life course, rather than an acute behavioural decision (23). By explicitly modelling total diabetes—capturing both diagnosed and undiagnosed disease—we also partially account for competing detection pathways (i.e., differential diagnosis and care contact) that may otherwise distort occupational risk estimates (24).

Although the cross-sectional design precludes temporal inference, the internal coherence of this pattern across multiple analytically independent outcomes suggests that it may reflect a relatively stable health–work equilibrium shaped by structural constraints, rather than a spurious statistical artifact.

### 4.2 The “sick labourer” phenotype: when occupational activity cannot function as metabolic therapy

A central insight of this study is that the metabolic implications of labour-type physical activity appear conditional rather than universal. Among older adults without diagnosed diabetes, frequent high-intensity labour was associated with a modest reduction in the triglyceride–glucose (TyG) index, a validated surrogate marker of systemic insulin resistance (25). However, the absolute magnitude of this reduction was limited, and TyG values remained elevated across all labour categories, suggesting that labour-related metabolic compensation in this setting may be insufficient once insulin resistance is established at the population level.

Importantly, TyG showed a strong and consistent association with diagnosed diabetes, exceeding the association observed for labour intensity. This pattern is compatible with a threshold-like interpretation: once systemic insulin resistance becomes substantial, the marginal glucose-disposing capacity of occupational exertion may be insufficient to restore metabolic homeostasis. This interpretation is offered as a conceptual explanation rather than a formally tested threshold model in the present cross-sectional data. A key physiological distinction from leisure-time exercise is that occupational physical activity (OPA) in this context often lacks adequate recovery periods, nutritional compensation, and autonomic downregulation, potentially shifting activity-related strain toward cumulative physiological stress rather than therapeutic adaptation (9).

The positive association between high-intensity labour and diagnosed diabetes further supports what we term the “Sick Labourer Phenotype”—a cross-sectional configuration consistent with reverse selection under livelihood constraint and distinct from the classical “healthy worker effect” (26). In labour-dependent rural settings, older adults with established metabolic disease may be unable to reduce workload despite illness and may continue high-intensity labour for household subsistence. Although we lacked direct measures of pension security and household economic dependency, this explanation is presented as hypothesis-generating and consistent with the context of labour-dependent rural ageing. In this framing, labour may function as an additional stressor superimposed on an already compromised metabolic system, potentially amplifying vulnerability to adverse outcomes rather than serving as metabolic therapy.

This interpretation remains inherently limited by the cross-sectional design and the use of a single frequency-based proxy for occupational exposure. Accordingly, the “Sick Labourer Phenotype” should be understood as a context-sensitive explanatory framework intended to organize observed associations and motivate longitudinal validation, rather than as evidence of temporality or causal inference.

### 4.3 Labour-associated UOB positivity as an exploratory urinary abnormality signal

Beyond metabolic outcomes, we identified an association between labour intensity and urine occult blood (UOB) positivity, interpreted here as a population-level, non-specific urinary abnormality signal compatible with physiological/urogenital stress rather than a disease-specific diagnosis. High-intensity labour was associated with a higher prevalence of UOB positivity in the primary modified Poisson models (PR 1.25; 95% CI 1.09–1.44), representing a modest effect size at the population level rather than a clinically diagnostic magnitude. Directionally consistent sensitivity estimates were observed under logistic regression (OR 1.39; 95% CI 1.13–1.70), although these are presented for comparative robustness only, given the tendency of odds ratios to overestimate prevalence associations in common outcomes.

UOB positivity is etiologically non-specific and may arise from transient or non-renal causes, including dehydration, cold exposure, exertional hematuria, urolithiasis (kidney stones), urinary tract infection or inflammation, and—among women—gynecologic sources of blood contamination (27, 28). Accordingly, we cannot localize the origin of UOB positivity within the urinary tract using the present dataset, and residual confounding by these conditions cannot be excluded. Nevertheless, the persistence and directional consistency of the association after multivariable adjustment makes it less likely that the signal is fully explained by random measurement noise alone, although non-renal etiologies and unmeasured confounding remain plausible.

Mechanistically, several hypotheses could link sustained high-intensity agricultural labour to a higher population prevalence of UOB positivity, including repeated hemodynamic strain under dehydration or cold stress, transient renal hypoperfusion, or exertion-related hematuria. These mechanistic pathways remain speculative and should not be interpreted as evidence of renal microvascular injury in the absence of confirmatory biomarkers. Without albuminuria/urine ACR, eGFR, or urine microscopy, we cannot distinguish glomerular from non-glomerular sources or separate renal from urogenital pathways. These findings should therefore be regarded as hypothesis-generating and warrant validation using more specific renal markers and longitudinal follow-up (29).

### 4.4 Contextualizing the findings: rural ageing in Northeast China

These findings should be interpreted within their specific socio-geographic context. Wangkui County represents a cold-climate, agriculture-dependent region where older adults often remain engaged in physically demanding work beyond conventional retirement age. In such settings, OPA differs fundamentally from leisure-time exercise in duration, intensity, recovery opportunity, and voluntariness (30–32).

Seasonal workload peaks, cold exposure, limited mechanization, and constrained access to labour substitution likely amplify cumulative physiological strain. These contextual factors plausibly shape both the metabolic offsetting pattern and the microvascular stress–related associations observed in this study, and limit direct extrapolation to urban or leisure-activity–dominant populations, where occupational workload is lower and physical activity is more discretionary.

In addition, rural-specific exposures that often co-vary with labour intensity—such as pesticide contact, extreme cold/heat exposure, dehydration risk, and socioeconomic disadvantage—may contribute to residual confounding (33, 34). These factors were not fully captured in the present dataset and may partly shape observed associations. Future work should incorporate explicit measures of agrochemical exposure, hydration/heat–cold stress indicators, and socioeconomic position to strengthen causal interpretability.

Importantly, the observed clustering of diagnosed diabetes within high-intensity labour groups must also be understood within the structural realities of rural ageing. In labour-dependent agricultural settings, continued engagement in physically demanding work is often driven not by health selection but by livelihood necessity. Limited pension coverage, inadequate retirement security, and household subsistence obligations may compel older adults to maintain heavy labour participation despite chronic illness (35, 36). Under such conditions, the classical “healthy worker effect” may be attenuated—or even reversed—giving rise to an economically constrained selection pattern in which metabolically compromised individuals remain embedded within high-intensity labour structures. This socio-medical context provides an important interpretive backdrop for the Sick Labourer Phenotype and reinforces the need to consider labour not solely as a behavioural exposure but as a structurally conditioned life-course constraint.

### 4.5 Implications for public health: from generic activation to “medical offloading”

Our findings suggest a needed recalibration of chronic disease prevention strategies for ageing, labour-dependent populations. Generic recommendations to “increase physical activity” may be inappropriate—or potentially harmful—when baseline occupational workload is already high and largely non-discretionary (37, 38).

We therefore propose “Medical Offloading”: a public health strategy aimed at reducing unavoidable physical burden as a form of disease prevention. Here, we define “Medical Offloading” as reducing non-discretionary occupational burden as a preventive complement to conventional activity-promotion strategies in labour-dependent older adults. For individuals exhibiting the Sick Labourer Phenotype (high labour intensity combined with elevated TyG), priorities may need to shift from further physical activation toward metabolic protection, workload redistribution, and social support mechanisms that reduce reliance on heavy manual labour (22, 39, 40).

Operationally, Medical Offloading may include three complementary pathways: (1) workload substitution through family, community, or mechanized labour support (40); (2) targeted metabolic risk screening and follow-up among high-labour older adults; and (3) seasonal risk mitigation strategies addressing dehydration, cold exposure, and peak agricultural workload periods (40). At a policy level, labour intensity can be conceptualized as a modifiable social exposure—analogous to occupational or environmental hazards—rather than an individual health behaviour, and integrated into rural ageing and chronic disease frameworks alongside metabolic risk screening and social protection measures.

### 4.6 Strengths, methodological boundaries, and future directions

This study is strengthened by the comprehensive definition of total diabetes burden, mechanistic integration of TyG, and coherent findings across multiple analytically independent regression models and sensitivity analyses (41–44). Nevertheless, causal inference is limited by the cross-sectional design, and residual confounding by unmeasured structural factors (e.g., detailed socioeconomic position, occupational history duration, and healthcare access) cannot be fully excluded.

Labour intensity was assessed using a standardised single-item, frequency-based self-report proxy and therefore cannot capture METs, duration, seasonality, recovery patterns, or cumulative occupational years. Such exposure misclassification is likely to be non-differential and would therefore bias associations toward the null rather than generate spurious positive findings (40). The very small moderate-intensity stratum limits dose–response inference; accordingly, we emphasised prespecified robustness analyses collapsing moderate and high labour into an “Active” category (Supplementary Tables S6–S10).

UOB is sensitive to physiological stress but lacks etiologic specificity; accordingly, the present findings should be interpreted as evidence of a labour-associated urinary abnormality signal rather than definitive renal pathology. Dipstick measurement variability, transient contamination, and unmeasured urogenital conditions remain plausible alternative explanations. Future longitudinal studies should incorporate albuminuria (urine ACR), eGFR, urine microscopy, tubular injury biomarkers (e.g., KIM-1, NGAL), and renal imaging to delineate causal pathways and phenotype the nature of labour-associated renal/urogenital stress processes (45–47). Finally, more granular characterization of occupational workload (intensity, seasonality, cumulative years, and recovery patterns) will be essential for translating “Medical Offloading” into scalable, context-appropriate interventions (48, 49).

Although the TyG index is mathematically derived from fasting plasma glucose and triglycerides, we strictly segregated its use to avoid mathematical coupling: TyG was included only in models of diagnosed diabetes (defined by self-reported diagnosis/medication history rather than contemporaneous FPG), and it was deliberately excluded from models where outcomes were defined using FPG (undiagnosed hyperglycemia) or composite definitions involving FPG (total diabetes).

### 4.7 Conceptual synthesis: labour as a life-course, context-dependent health exposure

TTaken together, these findings advance a conceptual reframing of labour in ageing public health research. In labour-dependent rural populations, heavy occupational workload is neither uniformly protective nor benign. Instead, it occupies a paradoxical position—providing limited metabolic compensation among individuals without diagnosed diabetes while disproportionately clustering among those with established disease, thereby cancelling any net protective effect at the population level. Simultaneously, heavy labour was associated with a non-specific urinary abnormality signal (UOB positivity) compatible with physiological/urogenital stress that is not fully captured by conventional cardiometabolic risk models. Importantly, this pattern suggests that occupational labour in later life should be understood not as a health behaviour, but as a life-course exposure shaped by structural constraint. In such contexts, labour intensity likely reflects cumulative occupational burden accrued over decades rather than a discretionary or health-motivated choice. Recognizing labour as a context-dependent biological stressor—rather than a universal proxy for healthy activity—is therefore essential for designing equitable, effective, and socially grounded interventions in rapidly ageing societies (18, 50).

The structural relationships synthesised here are consistent with the hypothesised conceptual framework derived from the empirical results (Figure 3); dashed links indicate hypothesised relationships intended to organise cross-sectional associations rather than to imply causal effects.

## 5 Conclusion

In a labour-dependent rural population of older adults, this study demonstrates that high-intensity occupational physical activity does not confer a net metabolic benefit and instead represents a paradoxical health exposure. While heavy labour is associated with a modest reduction in undiagnosed hyperglycemia among individuals without diagnosed diabetes, it disproportionately clusters among those with established disease, resulting in a cancellation of protective effects at the population level.

Simultaneously, high-intensity labour was independently associated with a higher prevalence of urine occult blood positivity, a population-level marker indicative of microvascular stress, irrespective of metabolic status.

These findings challenge the prevailing assumption that all forms of physical activity are universally beneficial in later life and emphasize the need to distinguish occupational labour from discretionary exercise. In ageing, labour-dependent settings, occupational physical activity should be understood not as a health behaviour, but as a life-course exposure shaped by structural and economic constraints. Under such conditions, sustained heavy labour may exceed the adaptive capacity of ageing physiology, potentially transforming exertion from a therapeutic stimulus into a source of chronic biological stress.

From a public health perspective, these results call for a fundamental recalibration of activity-based prevention strategies in rural ageing populations. Rather than uniformly promoting increased physical activity, interventions should prioritize “Medical Offloading”—the reduction of unavoidable physical burden—as a complementary strategy for metabolic and vascular protection. Recognizing labour intensity as a modifiable social exposure is essential for designing equitable, context-sensitive strategies that align healthy ageing goals with the lived realities of older adults in labour-dependent societies.

## Data Availability

The raw data supporting the conclusions of this article will be made available by the authors, without undue reservation.

## References

[1] Buchner DM. Physical activity and prevention of cardiovascular disease in older adults. Clin Geriatr Med. (2009) 25:661–75. doi: 10.1016/j.cger.2009.08.002, 19944266

[2] Saint-Maurice PF Troiano RP Bassett DR Graubard BI Carlson SA Shiroma EJ . Association of daily step count and step intensity with mortality among US adults. JAMA. (2020) 323:1151–60. doi: 10.1001/jama.2020.1382, 32207799 PMC7093766

[3] Holtermann A Krause N van der Beek AJ Straker L. The physical activity paradox: six reasons why occupational physical activity (OPA) does not confer the cardiovascular health benefits that leisure-time physical activity does. Br J Sports Med. (2018) 52:149–150. doi: 10.1136/bjsports-2017-097965, 28798040

[4] Stoner L Higgins S Paterson C. The 24-h activity cycle and cardiovascular outcomes: establishing biological plausibility using arterial stiffness as an intermediate outcome. Am J Physiol Heart Circ Physiol. (2023) 325:H1243–63. doi: 10.1152/ajpheart.00258.2023, 37737729 PMC11932535

[5] Cillekens B Lang M van Mechelen W Verhagen E Huysmans MA Holtermann A . How does occupational physical activity influence health? An umbrella review of 23 health outcomes across 158 observational studies. Br J Sports Med. (2020) 54:1474–81. doi: 10.1136/bjsports-2020-102587, 33239353

[6] Hu S-S. Report on cardiovascular health and diseases in China 2021: an updated summary. J Geriatr Cardiol. (2023) 20:399–430. doi: 10.26599/1671-5411.2023.06.001, 37416519 PMC10320777

[7] Zhou B Carrillo-Larco RM Danaei G Riley LM Paciorek CJ Stevens GA . Worldwide trends in hypertension prevalence and progress in treatment and control from 1990 to 2019: a pooled analysis of 1201 population-representative studies with 104 million participants. Lancet. (2021) 398:957–80. doi: 10.1016/S0140-6736(21)01330-1 34450083 PMC8446938

[8] Manne-Goehler J Geldsetzer P Agoudavi K Andall-Brereton G Aryal KK Bicaba BW . Health system performance for people with diabetes in 28 low- and middle-income countries: a cross-sectional study of nationally representative surveys. PLoS Med. (2019) 16:e1002751. doi: 10.1371/journal.pmed.1002751, 30822339 PMC6396901

[9] Holtermann A Krause N van der Beek AJ Straker L. The physical activity paradox: six reasons why occupational physical activity (OPA) does not confer the cardiovascular health benefits that leisure time physical activity does. Br J Sports Med. (2018) 52:149–50. doi: 10.1136/bjsports-2017-097965, 28798040

[10] Feuer DS Handberg EM Mehrad B Wei J Merz CNB Pepine CJ . Microvascular dysfunction as a systemic disease: a review of the evidence. Am J Med. (2022) 135:1059–68. doi: 10.1016/j.amjmed.2022.04.006, 35472396 PMC9427712

[11] Li Y Zhu L Yang Y Zhang C Zhao H Shi J . Perceived criticism and depressive symptoms among adults aged 50 years and older: a 17-year population-based cohort study. Transl Psychiatry. (2025) 15:178. doi: 10.1038/s41398-025-03322-6, 40410166 PMC12102303

[12] Aljafary MA Al-Suhaimi EA. Adiponectin system (rescue hormone): the missing link between metabolic and cardiovascular diseases. Pharmaceutics. (2022) 14:1430. doi: 10.3390/pharmaceutics14071430, 35890325 PMC9321059

[13] Akiboye RD Sharma DM. Haematuria in sport: a review. Eur Urol Focus. (2019) 5:912–6. doi: 10.1016/j.euf.2018.02.008, 29500137

[14] Sutton JM. Evaluation of hematuria in adults. JAMA. (1990) 263:2475–80. doi: 10.1001/jama.1990.03440180081037 2184261

[15] Sheikh K. A review of the healthy worker effect in occupational epidemiology. Occup Med (Lond). (2000) 50:146. doi: 10.1093/occmed/50.2.146, 10829439

[16] Arrighi HM Hertz-Picciotto I. The evolving concept of the healthy worker survivor effect. Epidemiology. (1994) 5:189–96. doi: 10.1097/00001648-199403000-00009, 8172994

[17] Kuh D Ben-Shlomo Y Lynch J Hallqvist J Power C. Life course epidemiology. J Epidemiol Community Health. (2003) 57:778–783. doi: 10.1136/jech.57.10.778 14573579 PMC1732305

[18] Marmot M Bell R. Social determinants and non-communicable diseases: time for integrated action. BMJ. (2019) 364:l251. doi: 10.1136/bmj.l251, 30692093 PMC6348404

[19] Elm E von Altman DG Egger M Pocock SJ Gøtzsche PC Vandenbroucke JP The strengthening the reporting of observational studies in epidemiology (STROBE) statement: guidelines for reporting observational studies. Lancet (2007) 370 1453–1457 doi: 10.1016/S0140-6736(07)61602-X 18064739

[20] Holtermann A Hansen JV Burr H Søgaard K Sjøgaard G. The health paradox of occupational and leisure-time physical activity. Br J Sports Med. (2012) 46:291–5. doi: 10.1136/bjsm.2010.079582 21459873

[21] Yu X Wang L Zhang W Ming J Jia A Xu S . Fasting triglycerides and glucose index is more suitable for the identification of metabolically unhealthy individuals in the Chinese adult population: a nationwide study. J Diabetes Investig. (2019) 10:1050–8. doi: 10.1111/jdi.12975, 30417578 PMC6626942

[22] Marmot M. Health equity in England: the Marmot review 10 years on. BMJ. (2020) 368:m693. doi: 10.1136/bmj.m693 32094110

[23] Lynch J Smith GD. A life course approach to chronic disease epidemiology. Annu Rev Public Health. (2005) 26:1–35. doi: 10.1146/annurev.publhealth.26.021304.144505, 15760279

[24] Prevett C Gingerich J Sivak A Davenport MH. Resistance training in pregnancy: systematic review and meta-analysis of pregnancy, delivery, fetal and pelvic floor outcomes and call to action. Br J Sports Med. (2025) 59:1173–82. doi: 10.1136/bjsports-2024-109123, 40610191

[25] Ye Z Xie E Jiao S Gao Y Li P Tu Y . Triglyceride glucose index exacerbates the risk of future cardiovascular disease due to diabetes: evidence from the China health and retirement longitudinal survey (CHARLS). BMC Cardiovasc Disord. (2022) 22:236. doi: 10.1186/s12872-022-02673-y, 35597912 PMC9124384

[26] Li CY Sung FC. A review of the healthy worker effect in occupational epidemiology. Occup Med (Lond). (1999) 49:225–9. doi: 10.1093/occmed/49.4.225, 10474913

[27] Munroe D O’Keefe J Wang D Moore MA. Evaluation of the 2020 American urological association microscopic Hematuria guidelines in clinical practice: retrospective chart review analysis. JMIR Form Res. (2025) 9:e75929. doi: 10.2196/75929, 41343761 PMC12677728

[28] Zhang L Zhang X Pu Y Zhang Y Fan J. Global, regional, and national burden of urolithiasis from 1990 to 2019: a systematic analysis for the global burden of disease study 2019. Clin Epidemiol. (2022) 14:971–83. doi: 10.2147/CLEP.S370591, 35996396 PMC9391934

[29] Battaglia Y Baciga F Bulighin F Amicone M Mosconi G Storari A . Physical activity and exercise in chronic kidney disease: consensus statements from the physical exercise working Group of the Italian Society of nephrology. J Nephrol. (2024) 37:1735–65. doi: 10.1007/s40620-024-02049-9, 39269600 PMC11519309

[30] Hu X Gu S Zhen X Sun X Gu Y Dong H. Trends in cognitive function among Chinese elderly from 1998 to 2018: an age-period-cohort analysis. Front Public Health. (2021) 9:753671. doi: 10.3389/fpubh.2021.753671, 34900900 PMC8660074

[31] Gasparrini A Guo Y Hashizume M Lavigne E Zanobetti A Schwartz J . Mortality risk attributable to high and low ambient temperature: a multicountry observational study. Lancet. (2015) 386:369–75. doi: 10.1016/S0140-6736(14)62114-0, 26003380 PMC4521077

[32] Parker HM Gallagher R Duffield C Ding D Sibbritt D Perry L. Occupational and leisure-time physical activity have different relationships with health: a cross-sectional survey study of working nurses. J Phys Act Health. (2021) 18:1495–502. doi: 10.1123/jpah.2020-0415, 34686623

[33] Tomic D Shaw JE Magliano DJ. The burden and risks of emerging complications of diabetes mellitus. Nat Rev Endocrinol. (2022) 18:525–39. doi: 10.1038/s41574-022-00690-7, 35668219 PMC9169030

[34] Park MY Kang M-Y. Occupational risk factors for kidney disease: a comprehensive review. J Korean Med Sci. (2025) 40:e224. doi: 10.3346/jkms.2025.40.e224, 40795345 PMC12339896

[35] Huang W Zhang C. The power of social pensions: evidence from China’s new rural pension scheme. Am Econ J Appl Econ. (2021) 13:179–205. doi: 10.1257/app.20170789

[36] Gu H Tan Q Guo Y He H Zhang Y. Family support, social security, commercial insurance, and aging anxiety among Chinese residents: a study based on the 2021 CGSS data. Front Public Health. (2025) 13:1577384. doi: 10.3389/fpubh.2025.1577384, 40265070 PMC12013337

[37] Bull FC Al-Ansari SS Biddle S Borodulin K Buman MP Cardon G . World Health Organization 2020 guidelines on physical activity and sedentary behaviour. Br J Sports Med. (2020) 54:1451–62. doi: 10.1136/bjsports-2020-102955, 33239350 PMC7719906

[38] Coenen P Huysmans MA Holtermann A Krause N van Mechelen W Straker LM . Do highly physically active workers die early? A systematic review with meta-analysis of data from 193 696 participants. Br J Sports Med. (2018) 52:1320–6. doi: 10.1136/bjsports-2017-098540, 29760168

[39] Anderson E Durstine JL. Physical activity, exercise, and chronic diseases: a brief review. Sports Med Health Sci. (2019) 1:3–10. doi: 10.1016/j.smhs.2019.08.006, 35782456 PMC9219321

[40] Holtermann A Mathiassen SE Straker L. Promoting health and physical capacity during productive work: the goldilocks principle. Scand J Work Environ Health. (2019) 45:90–7. doi: 10.5271/sjweh.3754, 30021029

[41] Zou G. A modified Poisson regression approach to prospective studies with binary data. Am J Epidemiol. (2004) 159:702–6. doi: 10.1093/aje/kwh090, 15033648

[42] Lee H Hwang J Yon DK Rhee SY. Multimodal and multidimensional artificial intelligence Technology in Obesity. J Obes Metab Syndr. (2025) 34:394–404. doi: 10.7570/jomes25035, 40922673 PMC12583783

[43] Topol EJ. High-performance medicine: the convergence of human and artificial intelligence. Nat Med. (2019) 25:44–56. doi: 10.1038/s41591-018-0300-7, 30617339

[44] Irvin L Madden LA Marshall P Vince RV. Digital health solutions for weight loss and obesity: a narrative review. Nutrients. (2023) 15:1858. doi: 10.3390/nu15081858, 37111077 PMC10145832

[45] Obert LA Elmore SA Ennulat D Frazier KS. A review of specific biomarkers of chronic renal injury and their potential application in nonclinical safety assessment studies. Toxicol Pathol. (2021) 49:996–1023. doi: 10.1177/0192623320985045, 33576319 PMC8195817

[46] Greenberg JH Abraham AG Xu Y Schelling JR Feldman HI Sabbisetti VS . Urine biomarkers of kidney tubule health, injury, and inflammation are associated with progression of CKD in children. J Am Soc Nephrol. (2021) 32:2664–77. doi: 10.1681/ASN.2021010094, 34544821 PMC8722795

[47] Caroli A Pruijm M Burnier M Selby NM. Functional magnetic resonance imaging of the kidneys: where do we stand? The perspective of the European COST action PARENCHIMA. Nephrol Dial Transplant. (2018) 33:ii1–3. doi: 10.1093/ndt/gfy181, 30137582 PMC6106640

[48] Mabweazara SZ Leach LL Ley C. Development of a context-sensitive physical activity intervention for persons living with HIV and AIDS of low socioeconomic status using the behaviour change wheel. BMC Public Health. (2019) 19:774. doi: 10.1186/s12889-019-7091-8, 31208375 PMC6580554

[49] Straker L Mathiassen SE Holtermann A. The ‘goldilocks principle’: designing physical activity at work to be ‘just right’ for promoting health. Br J Sports Med. (2018) 52:818–9. doi: 10.1136/bjsports-2017-097765, 28663212 PMC6029635

[50] Holtermann A Schnohr P Nordestgaard BG Marott JL. The physical activity paradox in cardiovascular disease and all-cause mortality: the contemporary Copenhagen general population study with 104 046 adults. Eur Heart J. (2021) 42:1499–511. doi: 10.1093/eurheartj/ehab087, 33831954 PMC8046503

