# Supplementary material for: The “Futile Labour” Paradox: occupational physical activity fails to offset diabetes burden and is associated with microvascular stress signals in rural older adults
Source: Front Public Health. 2026 Mar 16;14:1790766. doi: 10.3389/fpubh.2026.1790766 (PMC13033493; doi:10.3389/fpubh.2026.1790766)
Supplement: Supplementary file 1 [file Table_1.docx]

**Supplementary Table S1. Sample characteristics and diabetes burden after data cleaning**

| **Metric** | **Value** |
| --- | --- |
| Final analytic sample size | 2,258 |
| Participants with total diabetes, n (%) | 519 (22.98%) |
| Diagnosed diabetes, n (%) | 281 (12.44%) |
| Undiagnosed hyperglycemia among eligible participants, n (%) | 272 / 1,904 (14.29%) |
| Participants without diagnosed diabetes (eligible population) | 1,904 |

**Footnote:** Total diabetes was defined as self-reported physician-diagnosed diabetes or fasting plasma glucose (FPG) ≥ 7.0 mmol/L. Undiagnosed hyperglycemia was assessed only among participants without self-reported diabetes or hypoglycemic medication use (eligible population, n = 1,904).

**Supplementary Table S2. Sex-stratified sensitivity analyses: logistic regression odds ratios for urine occult blood (UOB) positivity**

**Men (complete-case n = 1,058)**

| **Variable** | **OR** | **95% CI** | **P value** |
| --- | --- | --- | --- |
| Moderate vs. Inactive | 1.04 | 0.68–1.59 | 0.861 |
| High vs. Inactive | 1.46 | 1.06–2.01 | 0.021 |

**Women (complete-case n = 1,123)**

| **Variable** | **OR** | **95% CI** | **P value** |
| --- | --- | --- | --- |
| Moderate vs. Inactive | 0.97 | 0.63–1.49 | 0.893 |
| High vs. Inactive | 1.28 | 0.97–1.69 | 0.076 |

**Footnote:** Sex-stratified logistic regression models were fitted as sensitivity analyses to complement the primary PR estimates (Supplementary Table S12). Models adjusted for age, BMI, hypertension, systolic blood pressure (SBP), diabetes status, TyG index (when applicable), smoking, drinking, and diet type (where available). Urine dipstick protein was additionally adjusted for where available.

**Supplementary Table S3. Sensitivity analysis: Diagnosed diabetes excluding BMI**

| **Variable** | **PR** | **95% CI** | **P value** | **n** |
| --- | --- | --- | --- | --- |
| Moderate vs. Inactive | 0.33 | 0.08–1.36 | 0.119 | 2,181 |
| High vs. Inactive | 1.44 | 1.11–1.87 | 0.006 | 2,181 |

**Footnote:** Modified Poisson regression with robust (HC3) standard errors. BMI was excluded to evaluate potential mediation by adiposity. Models adjusted for age, sex, hypertension, SBP, smoking, drinking, and diet type (where available). Analyses were conducted in the complete-case subset (n = 2,181).

**Supplementary Table S4. Sensitivity analysis: Undiagnosed hyperglycemia excluding BMI**

| **Variable** | **PR** | **95% CI** | **P value** | **n** |
| --- | --- | --- | --- | --- |
| Moderate vs. Inactive | 1.19 | 0.61–2.32 | 0.603 | 1,904 |
| High vs. Inactive | 0.75 | 0.57–0.98 | 0.030 | 1,904 |

**Footnote:** Modified Poisson regression with robust (HC3) standard errors among participants without diagnosed diabetes. BMI was excluded to evaluate potential mediation by adiposity. TyG index was not included to avoid definitional overlap, because undiagnosed hyperglycemia was defined using fasting plasma glucose.

**Supplementary Table S5. Trend test for labour intensity and total diabetes burden**

| **Variable** | **PR per category increase** | **95% CI** | **P value** | **n** |
| --- | --- | --- | --- | --- |
| Labour intensity (ordinal) | 0.99 | 0.88–1.12 | 0.946 | 2,181 |

**Footnote:** Labour intensity was modeled as an ordinal variable (Inactive = 1, Moderate = 2, High = 3). Modified Poisson regression with robust (HC3) standard errors adjusted for age, sex, BMI, hypertension, SBP, smoking, drinking, and diet type (where available). TyG index was excluded to avoid mathematical circularity where outcomes were defined using contemporaneous FPG.

**Supplementary Table S6. Exposure reclassification cross-tabulation (Ex_Cat × Ex_Bin)**

| **Original category** | **Binned category** | **Frequency** |
| --- | --- | --- |
| Inactive | Inactive | 845 |
| Moderate | Active | 46 |
| High | Active | 1,312 |
| Missing | Missing | 55 |

**Footnote:** Exposure reclassification merged Moderate and High categories into an “Active” stratum to stabilize inference under small-cell conditions. Cross-tabulation verified correct binning. Participants with missing exposure (n = 55) were excluded from complete-case regression models.

**Supplementary Table S7. Diagnosed diabetes — Ex_Bin robustness (Base model; n = 2,181)**

| **Variable** | **PR** | **95% CI** | **P value** |
| --- | --- | --- | --- |
| Active vs. Inactive | 1.38 | 1.07–1.79 | 0.014 |
| Age | 0.95 | 0.93–0.97 | <0.001 |
| Sex | 0.83 | 0.66–1.04 | 0.102 |
| BMI | 1.02 | 0.98–1.05 | 0.336 |
| Hypertension | 5.97 | 4.31–8.28 | <0.001 |
| SBP | 0.99 | 0.99–1.00 | 0.012 |

**Footnote:** Modified Poisson regression with robust (HC3) standard errors. “Active” combines Moderate and High labour categories. Covariates follow the corresponding primary base-model specification.

**Supplementary Table S8. Diagnosed diabetes — Ex_Bin mechanistic model (n = 2,181)**

| **Variable** | **PR** | **95% CI** | **P value** |
| --- | --- | --- | --- |
| Active vs. Inactive | 1.39 | 1.08–1.80 | 0.012 |
| TyG index | 1.97 | 1.68–2.30 | <0.001 |
| Resting heart rate | 1.00 | 0.99–1.01 | 0.998 |

**Footnote:** Modified Poisson regression with robust (HC3) standard errors. Covariates follow the corresponding primary mechanistic-model specification.

**Supplementary Table S9. Undiagnosed hyperglycemia — Ex_Bin robustness analysis (Active vs. Inactive; n = 1,904)**

| Variable | PR | 95% CI | P value |
| --- | --- | --- | --- |
| Active vs. Inactive | 0.71 | 0.55–0.91 | 0.007 |
| Age, years | 1.00 | 0.98–1.02 | 0.959 |
| Sex (Male vs Female) | 1.48 | 1.14–1.92 | 0.003 |
| BMI, kg/m² | 1.09 | 1.06–1.13 | <0.001 |
| Hypertension (Yes vs No) | 1.20 | 0.93–1.55 | 0.172 |
| SBP, mmHg | 1.00 | 1.00–1.01 | 0.565 |
| Smoking status | 1.05 | 0.72–1.53 | 0.793 |
| Drinking frequency | 0.97 | 0.81–1.16 | 0.704 |

**Footnote:** Modified Poisson regression with robust (HC3) standard errors. “Active” combines Moderate and High labour intensity categories to stabilize inference under small-cell conditions. Models were adjusted for age, sex, BMI, hypertension, SBP, smoking status, and drinking frequency. Consistent with the primary analysis, TyG index was not included to avoid definitional overlap with the fasting plasma glucose–defined outcome. All estimates represent cross-sectional associations and do not imply causality.

**Supplementary Table S10. Total diabetes — Ex_Bin robustness analysis (n = 2,181)**

| **Variable** | **PR** | **95% CI** | **P value** |
| --- | --- | --- | --- |
| Active vs. Inactive | 0.98 | 0.83–1.16 | 0.843 |

**Footnote:** Modified Poisson regression with robust (HC3) standard errors. “Active” combines Moderate and High labour intensity categories. Estimates were directionally consistent with the corresponding primary total-diabetes model.

**Supplementary Table S11. Multicollinearity diagnostics (GVIF)**

| **Variable** | **GVIF_adj** |
| --- | --- |
| Exposure category | 1.03 |
| Resting heart rate | 1.00 |
| Age | 1.05 |
| Sex | 1.04 |
| BMI | 1.05 |
| Hypertension | 1.04 |
| SBP | 1.04 |
| Smoking | 1.05 |
| Drinking | 1.07 |

**Footnote:** GVIF_adj denotes GVIF^(1/(2×df)) computed from the mechanistic-model covariate set fitted in the complete-case subset (zero-variance covariates removed). Values <2 were considered acceptable, indicating no evidence of problematic multicollinearity.

**Supplementary Table S12. UOB positivity — Modified Poisson PR model (primary; n = 2,169)**

| **Variable** | **PR** | **95% CI** | **P value** |
| --- | --- | --- | --- |
| High vs. Inactive | 1.25 | 1.09–1.44 | 0.002 |
| Moderate vs. Inactive | 1.00 | 0.59–1.68 | 0.999 |

**Footnote:** Modified Poisson regression with robust (HC3) standard errors. Models adjusted for age, sex, BMI, hypertension, SBP, diabetes status, TyG index (when applicable), smoking, drinking, diet type (where available), and urine dipstick protein (where available). P values are rounded to three decimals (e.g., 0.0018 reported as 0.002). TyG index was included in the complete-case subset where TG and FPG were available.

**Supplementary Table S13. OR versus PR for UOB positivity (effect-measure comparison)**

| **Contrast** | **OR (logistic)** | **PR (modified Poisson)** | **Relative difference (OR vs PR)** |
| --- | --- | --- | --- |
| High vs. Inactive | 1.39 | 1.25 | +11.2% |

**Footnote:** For common outcomes, ORs can be numerically farther from the null than PRs even when fitted to the same data. This table provides a transparent, single-contrast comparison between the logistic-regression OR and the modified-Poisson PR used in the main analyses.

**Supplementary Table S14. Firth penalized logistic regression robustness check for UOB positivity**

| **Variable** | **OR** | **95% CI** | **P value** |
| --- | --- | --- | --- |
| High vs. Inactive | 1.38 | 1.13–1.70 | 0.002 |

**Footnote:** Firth penalized logistic regression was used as an additional robustness check to mitigate small-cell bias. Estimates are shown for the primary contrast (High vs. Inactive).
